# Clinical efficacy of transforaminal endoscopic lumbar discectomy for lumbar degenerative diseases: A minimum 6-year follow-up

**DOI:** 10.3389/fsurg.2022.1004709

**Published:** 2022-09-15

**Authors:** Jin Tang, Ying Li, Congjun Wu, Wei Xie, Xugui Li, Xuewen Gan, Qilin Lu

**Affiliations:** ^1^ Department of Orthopaedics, Hubei 672 Orthopaedics Hospital of Integrated Chinese / Western Medicine, Wuhan, China; ^2^ Wuhan Sports University, Wuhan, China

**Keywords:** transforaminal endoscopic lumbar discectomy (TELD), lumbar degenerative disease (LDDs), disc height (DH), disc range of motion (ROM), pfirrmann grade, postoperative dysesthesia (POD)

## Abstract

**Background:**

Transforaminal Endoscopic Lumbar Discectomy (TELD) is widely applied for lumbar degenerative disease (LDDs) and satisfactory short-term outcomes have been achieved. However, the mid-term and long-term follow-up of this technique is still lacking.

**Objective:**

To retrospectively analyze the mid-term clinical efficacy of TELD for single-level LDD and its effect on intervertebral disc degeneration with a minimum of 6-year follow-up.

**Methods:**

64 patients with single-level LDDs (lumbar disc herniation, lumbar spinal stenosis) who underwent TELD under local anesthesia in our department from December 2014 to December 2015 were observed. Visual analog scale (VAS), Japanese Orthopaedic Association evaluation treatment (JOA) score and Oswestry Disability Index (ODI) were calculated and compared before operation, 3 months after operation, 6 months after operation, 1 year after operation and at the last follow-up. Disc Height (DH), disc range of motion (ROM) and disc degeneration on standard lumbar lateral radiographs before operation and at the last follow-up were determined. Recurrence rate and operation-related complications during follow-up were recorded.

**Results:**

64 cases were followed up for 6.4 ± 0.1 years. There were no complications such as infection, epidural hematoma and nerve root injury. 1 patient (1.67%) was found to have dural rupture and cauda equina hernia during the operation. There were significant differences in VAS, JOA, ODI between preoperative and postoperative 3 months, 6 months, 1 year and last follow-up (*P* < 0 01), VAS, JOA, ODI at 3 months after operation were different from 6 months after operation (*P* < 0 05), and there were significant differences compared with preoperative, 1 year after operation and last follow up (*P* < 0 01). VAS, JOA and ODI at 6 months after operation were significantly different from those before operation (*P* < 0.01), but not significantly different from those at 1 year after operation and the last follow-up (*P* > 0.05). There was no significant difference in DH, ROM and the Pfirrmann grade of intervertebral disc preoperative and the last follow-up. During the follow-up period, 3 patients (4.69%) were recurrent, 13 patients (20.31%) had various degrees of postoperative dysesthesia (POD), and 3 patients (4.69%) had various degrees of muscle weakness.

**Conclusion:**

TELD has a satisfactory mid-term efficacy, and has no significant effect on the DH, the stability of the intervertebral disc space, or on intervertebral disc degeneration. However, as expected, TELD was associated with some complications including recurrent disc herniation and POD.

## Introduction

Lumbar degenerative diseases (LDDs), one of the most common orthopedic issues, is associated with a morbidity of 20%–35% typically in populations aged older than 50 years (1, 2). Approximately 40%–60% of these patients merit a surgical intervention when conservative management has failed (3). Surgical procedures utilized to treat LDDS include traditional open discectomy (OD), microdiscectomy (MD), and more recently the minimally invasive techniques including percutaneous endoscopic discectomy (PED) and microendoscopic discectomy (MED) (4). With the development of minimally invasive technology, percutaneous endoscopic surgery has gradually become an important surgical approach for LDDs, with the transforaminal approach being the most widely utilized minimally invasive approach for lumbar discectomy (5, 6). The indications for transforaminal endoscopic lumbar discectomy (TELD) range from the original lumbar disc herniation (LDH) (7) to the present lumbar stenosis (LSS) (8), lumbar vertebral metastasis (9), lumbar discal cyst (10), and recurrent lumbar disc herniation (11).

At present, there are many studies on the treatment of LDD by transforaminal endoscopic lumbar discectomy (TELD), but most of these studies evaluated preoperative indications and assessed short-term follow-up without evaluating mid- and long-term outcomes. Mid and long-term follow-up has been reported in only a few studies (12, 13).

To retrospectively analyze the mid-term clinical efficacy of TELD for single-level LDD and its effect on intervertebral disc degeneration, we retrospectively analyzed 64 patients with LDD treated with TELD in our hospital from December 2014 to December 2015 with a follow-up of at least 6 years. The relevant data were sorted and analyzed as follows:

## Materials and methods

### General data

From December 2014 to December 2015, 75 patients with single segmental LDDs (lumbar disc herniation, lumbar stenosis) who underwent TELD in our hospital were selected as the study subjects. 11 cases were detached, 2 cases refused a hospital visit, 7 cases lost contact, 2 cases died of medical diseases. Finally, 64 cases who met the inclusion and exclusion criteria were enrolled and followed for at least 6 years.

### Inclusion criteria:

(1) A diagnosis of LDH (zone 3 or 4) (14) or LSS (lateral recess or foraminal stenosis) based on the patient's medical history, signs and imaging, with the involved segment clearly defined as a single segment;
(2) Unsatisfactory results after systematic conservative treatment for more than 3 months;
(3) The onset is not long, but the pain is severe and severely affects daily life, or there is significant disability and neurological deficit;
(4) No previous lumbar surgery history;
(5) No obvious surgical contraindications;
(6) Patients and their families had good compliance and were willing to cooperate with treatment and follow visits;
(7) Follow-up data was complete.

### Exclusion criteria

(1) Previous lumbar surgery;
(2) Patients with spondylolysis, central spinal canal stenosis, severe lumbar instability, lumbar spondylolisthesis, and tumors in the lumbar spinal canal;
(3) Multi-segmental lesions, the involved segment could not be defined as a single segment;
(4) L5/S1 disc herniation in patients where a superiorly located iliac crest made the transforaminal approach impossible;
(5) Patients or their families had poor compliance and were unwilling to cooperate with treatment and follow up visits;
(6) Patients with a history of psychological disorders;
(7) Follow-up data was incomplete.

### Surgical methods

The operation was performed by senior doctors in the same group. The TESSYS endoscopic spinal surgery system (endoscope, 18G puncture needle, soft tissue dilatation tube, working channel, nucleus pulposus forceps and blue forceps, etc.) produced by Joimax Company in Germany, and an Elliquence disposable radiofrequency plasma operation electrode were used. All patients underwent a transforaminal approach in the prone position under local anesthesia. After conventional catheterization, the diseased intervertebral foramen was resected step by step with a ring saw under fluoroscopy, and part of the ventral superior articular process was resected. Patients with spinal stenosis need to enlarge the ventral superior articular process. The operation was conducted utilizing the endoscope. Care was taken to avoid injury to the dura mater, nerve roots, and intervertebral endplates. The ligaments and small joints were preserved as much as possible, and sequestered nucleus pulposus and nucleus pulposus that caused symptoms were removed thoroughly. For patients with spinal stenosis, the starting point and stop point of ligamentum flavum were exposed and removed completely. Simultaneously, thermal annuloplasty was carried out until the dura mater and nerve roots were decompressed completely. We used bipolar to promote fibrosis of the annulus fibrosis after removal of the nucleus pulposus to prevent the reoccurrence of the LDH. The wound was closed with a stitch after complete hemostasis under the endoscope. A drainage tube was placed in case of excessive bleeding (After decompression, the normal saline perfusion was turned off, and all the blood oozed under the endoscope) (15). The drainage tube could be removed 1–2 days after the operation depending on the amount of drainage.

### Observation index

**Figure 1:**
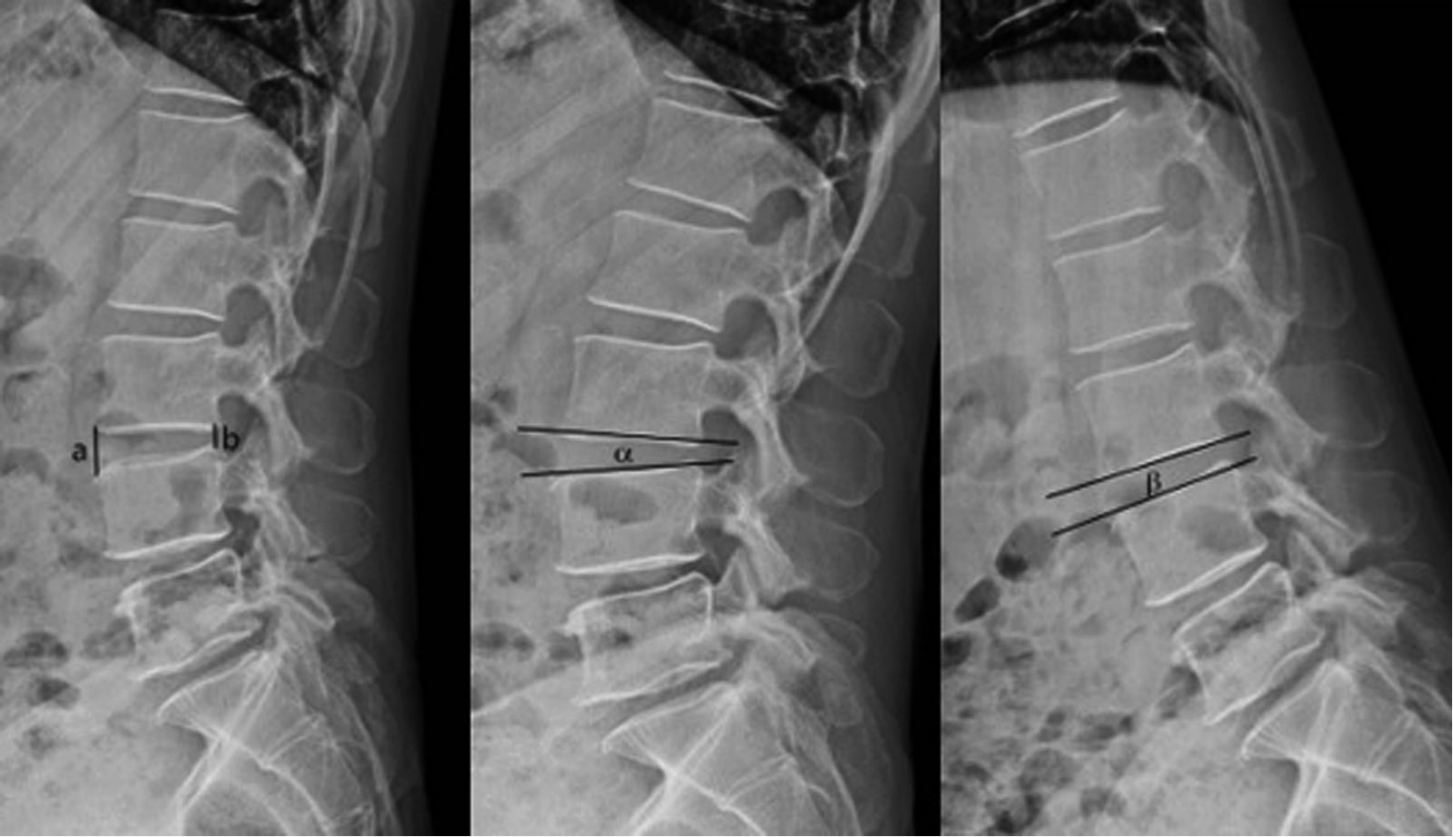
Dh and ROM: DH measurement method: the average value of the sum of the lines of the anterior and posterior edge of the upper and lower vertebral body, DH = (a + b)/2. ROM measurement method: the difference between the angle of intervertebral space in hyperextended position and flexion position, ROM = *α*-*β*.

(1) Clinical outcome: The visual analog scale (VAS) was adopted to assess the leg pain preoperation (1 day prior to surgery), as well as 3 months, 6 months, 1 year after surgery and the last follow-up. The Japanese Orthopaedic Association (JOA) score was adopted to assess the neurological function of patients preoperation (1 day prior to surgery), as well as 3 months, 6 months, 1 year after surgery and the last follow-up. The Oswestry Disability Index (ODI) was adopted to assess the low back pain preoperation (1 day prior to surgery), as well as 3 months, 6 months, 1 year after surgery and the last follow-up.
(2) Radiological outcome: disc height (DH) and range of motion (ROM) (Figure 1) were obtained preoperation and at the last follow-up by the lumbar x-ray radiographs in the anteroposterior and lateral position as well as dynamic position, to observe whether there was any intervertebral instability. ROM > 10° was defined as lumbar instability (16). The lumbar MRI was conducted preoperation and at the last follow-up to observe the degree of intervertebral disc degeneration (17). See Table 1 for details.
(3) Recurrence rate: the proportion of patients with ipsilateral recurrence of the same segment during the follow-up period (Recurrence rate = Recurrence cases/total cases × 100%).
(4) Incidence rate of adjacent segment disease (ASD): the proportion of patients with ASD after TELD (Incidence rate = ASD cases/ total cases × 100%).
(5) Operation-related complications: nerve injury, infection, dural rupture, postoperative dysesthesia (POD).

**Table 1:** Modified pfirrmann grade of disc degeneration (16).

Grade	Signal from nucleus and inner fibers of anulus	Distinction between inner and outer fibers of anulus at posterior aspect of disc	Height of disc
	Uniformly hyperintense, equal to CSF	Distinct	Normal
2	Hyperintense (>presacral fat and <CSF) ± hypointense intranuclear cleft	Distinct	Normal
3	Hyperintense though <presacral fat	Distinct	Normal
4	Mildly hyperintense (slightly > outer fibers of anulus)	Indistinct	Normal
5	Hypointense (= outer fibers of anulus)	Indistinct	Normal
6	Hypointense	Indistinct	<30% reduction in disc height
7	Hypointense	Indistinct	30%–60% reduction in disc height
8	Hypointense	Indistinct	>60% reduction in disc height

### Postoperative management

All patients received routine anti-infection (for 48 h) and symptomatic treatments after surgery. Patients were allowed to get out of bed to perform moderate activity for 15–30 min under the protection of the waistline at 24 h after surgery. After the symptoms relieved, patients were instructed to perform straight leg raising and lower back exercises. Improper waist postures, such as sitting or standing for a long time, bending down, and weight bearing, were avoided. A full rest was taken for three months, and physical labor was avoided within half a year.

### Statistical analysis

Measurement data are expressed as the mean ± standard deviation. All data were analyzed *via* SPSS 23.0 software. Count data were compared with the chi-squared test. The independent sample F test was used for intergroup comparisons. *P* < 0.05 was considered statistically significant, and *P* < 0.01 was deemed highly significant.

## Results

### General condition

**Figure 2:**
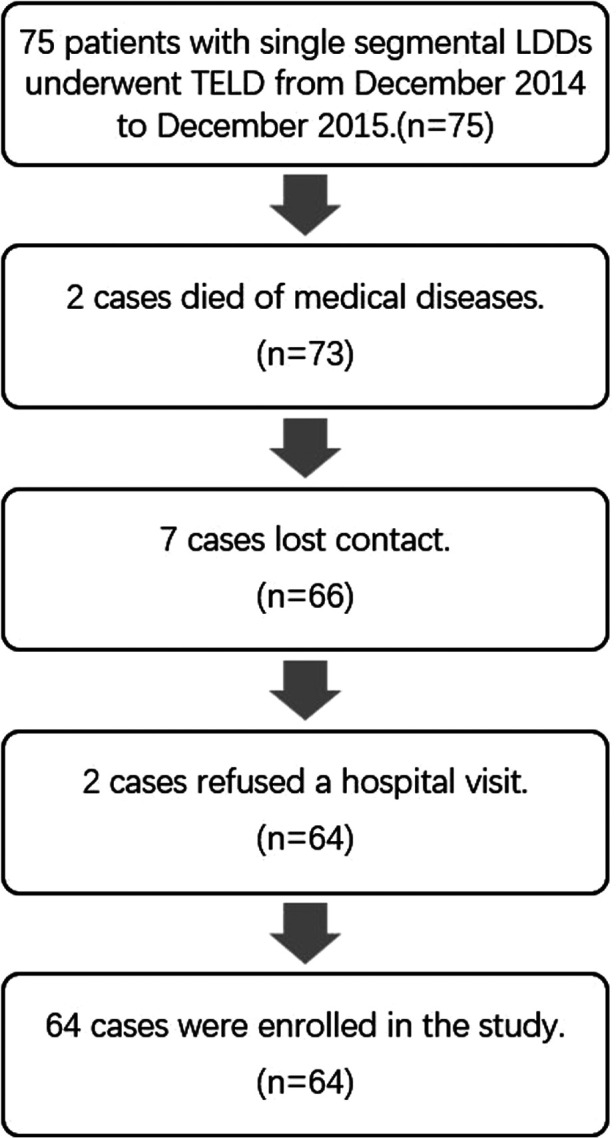
Flow chart.

All the surgeries were performed smoothly, and the complete data of 64 cases were included in this research (The flow chart is shown in Figure 2). The baseline demographic data of patients are shown in Table 2.

**Table 2:** Baseline demographic data of patients.

	Gender		Average Age (year)	Disease type			Surgery section		
	Male	Female		LDH	LSS	L2/3	L3/4	L4/5	L5/S1
Cases	28	36	57.72 ± 18.31	49	15	3	8	34	19

### Clinical outcome

There were significant differences in VAS, JOA, and ODI preoperative and postoperative values at 3 months, 6 months, 1 year and last follow-up (*P* < 0 01). The VAS, JOA, and ODI scores 3 months after the operation were significantly different from those 6 months after the operation (*P* < 0 05), and there were significant differences compared with values preoperatively and at the 1-year and last follow-ups (*P* < 0 01). The VAS, JOA and ODI at 6 months after the operation were significantly different from those before the operation (*P* < 0.01) but not significantly different from those at 1 year and the last follow-up (*P* > 0.05). See Table 3 for details.

**Table 3:** Comparison of preoperative and postoperative VAS, JOA and ODI.

	Preoperative	Postoperative 3 months	Postoperative 6 months	Postoperative 1 years	Last follow-up	F	*P*
VAS	8.64 ± 0.97	2.13 ± 1.28	1.69 ± 1.24	1.55 ± 1.08	1.45 ± 1.08	490.68	0
JOA	7.56 ± 4.25	22.22 ± 3.84	23.63 ± 3.83	24.11 ± 3.51	24.58 ± 2.91	245.84	0
ODI	71.86 ± 12.98	14.03 ± 6.12	11.48 ± 4.67	10.59 ± 3.75	10.03 ± 3.37	923.273	0

### Radiological outcome

DH was 10.22 ± 0.65 mm before the operation and 10.19 ± 0.66 mm at the last follow-up, but there was no significant difference between them (*P *> 0.05). The ROM was 5.59 ± 2.22° before the operation and 5.41 ± 2.31° at the last follow-up, but there was no significant difference between them (*P *> 0.05). See Table 4 for details.

**Table 4:** Comparison of preoperative and postoperative DH and ROM.

	Preoperative	Last follow-up	*t*	*P*
DH (mm)	10.22 ± 0.65	10.19 ± 0.66	0.211	0.833
ROM (°)	5.59 ± 2.22	5.41 ± 2.31	0.469	0.640

Before the operation, there were 4 patients with Pfirrmann grade 3, 6 patients with grade 4, 5 patients with grade 5, 6 patients with grade 6, 10 patients with grade 7 and 33 patients with grade 8. At the last follow-up, there were 1 patient with Pfirrmann grade 3, 2 patients with grade 4, 3 patients with grade 5, 5 patients with grade 6, 11 patients with grade 7 and 42 patients with grade 8. There was no significant difference between them (*P *> 0.05). See Table 5 for details.

**Table 5:** Comparison of preoperative and postoperative Modified Pfirrmann Grade.

	1	2	3	4	5	6	7	8
Preoperative	0	0	4	6	5	6	10	33
Last follow-up	0	0	1	2	3	5	11	42
X^2^	5.519							
*P*	0.356							

### Recurrence rate

During the follow-up period, 3 patients (4.69%) had lower limb root pain caused by ipsilateral intervertebral disc herniation (they were not excluded in the result calculations), 1 case appeared after bending down to carry heavy objects approximately 1 month after operation, 2 cases returned to normal work (sedentary station for a long time). The symptoms of 1 case relieved gradually after 2 weeks of conservative treatment. 1 case underwent endoscopic surgery again, 1 case underwent Mis-TLIF operation, and there were no symptoms in the last follow-up.

### Incidence rate of ASD

There was 1 patient (1.56%) with L3/4 spinal stenosis more than 3 years after L4/5 TELD, and TLIF surgery was performed after 3 months of conservative treatment without significant relief of symptoms. The postoperative symptoms disappeared, and no obvious symptoms occurred at the end of follow-up.

### Operation-related complications

No patients experienced postoperative infection, epidural hematoma, or nerve root injury. 1 patient (1.56%) was found to have dural rupture and cauda equina hernia during the operation. We gave head low and feet high position (raising the bed tail approximately 10–15 cm), used antibiotics that can pass through the blood-brain barrier to prevent the occurrence of intracranial infection, and strengthened fluid supplementation for him. No obvious spinal fluid leakage was observed after drainage tube placement (It was removed after 2 days), and no obvious symptoms related to cerebrospinal fluid leakage were found in the patient. Up to the last follow-up, there were no obvious symptoms. Thirteen patients (20.31%) showed various degrees of POD, all of them were exiting nerve root symptoms, were given acupuncture, medium-frequency pulse and neurotrophic drug for 3 months, and all of them recovered within 3 months after the operation, and 3 patients (4.69%) showed various degrees of muscle weakness (from grade 4 preoperatively to grade 3 postoperatively) and completely returned to grade 5 within 6 months after physiotherapy, such as neurotrophic drug and acupuncture.

#### Typical case 1 normal TELD patient

**Figure 3:**
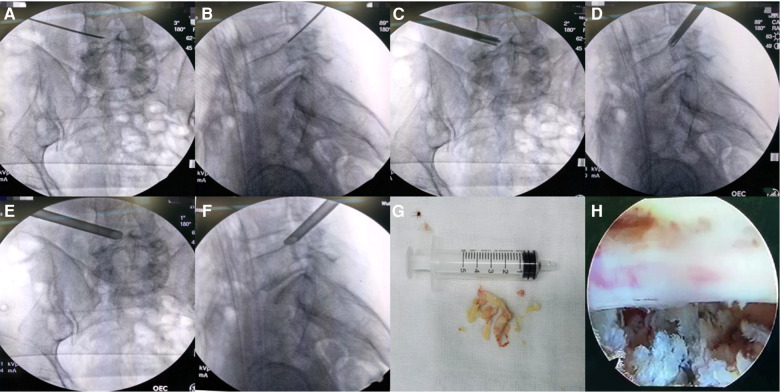
(**A,B**) anteroposterior and lateral position x-ray radiographs indicate the location of the spinal needle; (**C,D**) anteroposterior and lateral position x-ray radiographs indicate the location of the serrated reamer, which was used for zygapophyseal plasty; **(E,F**) anteroposterior and lateral position x-ray radiographs indicate the location of the working channel; (**G**) extracted the prolapse of nucleus pulposus; (**H**) sufficient decompression of the nerve root was seen under the endoscope.

**Figure 4:**
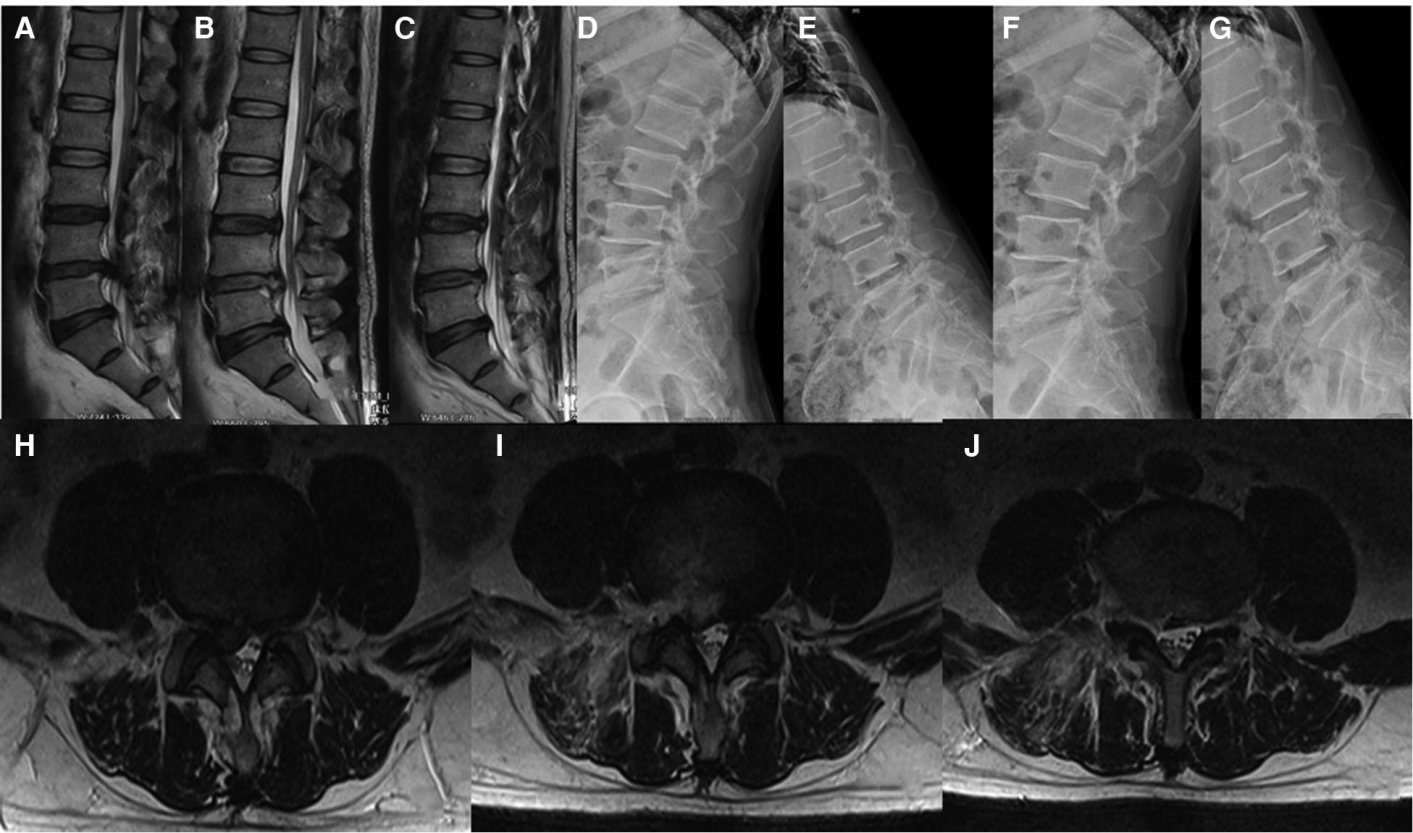
(**A**) preoperative MRI in sagittal position; (**B**) sagittal MRI 2 days after surgery; (**C**) sagittal MRI 6.5 years after surgery; (**D,E**) preoperative x-ray radiograph in lumbar dynamic position showing no lumbar instability or spondylolysis; (**F,G**) x-ray radiograph in lumbar dynamic position 6.5 years after surgery showing no lumbar instability;(**H**) preoperative MRI in coronal position; (**I**) coronal MRI 2 days after surgery; (**J**) coronal MRI 6.5 years after surgery.

Figures 3, 4 show the radiographs and MRI, respectively, of a representative 51-year-old male patient with right leg pain who was treated with TELD.

#### Typical case 2 recurrence after TELD

**Figure 5:**
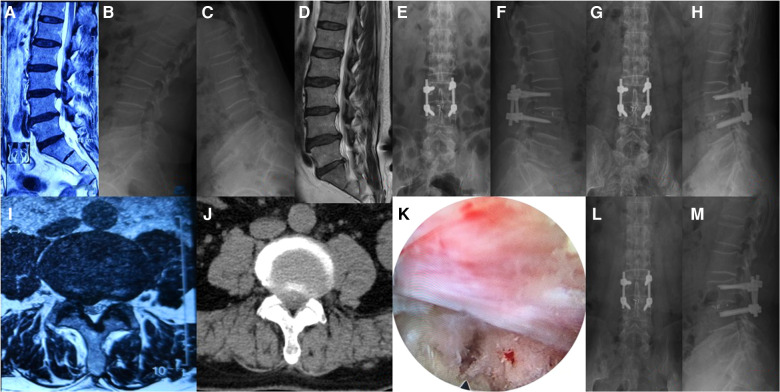
(**A**) preoperative MRI in sagittal position; (**B,C**) preoperative x-ray radiograph in lumbar dynamic position showing no lumbar instability or spondylolysis;(**D**) sagittal MRI 6 months after surgery; (**E,F**) anteroposterior and lateral position x-ray radiographs 3 days after Mis-TLIF; (**G,H**) anteroposterior and lateral position x-ray radiographs 3 months after Mis-TLIF;(**I**) preoperative MRI in coronal position; (**J**) preoperative CT in coronal position; (**K**) sufficient decompression of the nerve root was seen under the endoscope; (**L,M**) anteroposterior and lateral position x-ray radiographs 6 years after Mis-TLIF.

A 60-year-old male patient was admitted to the hospital with right leg pain for more than 3 months. After L3/4 TELD, the symptoms were completely relieved. 6 months after surgery, severe pain in the right leg recurred. The patient refused TELD and we performed Mis-TLIF. At the end of follow-up, the patient did not show any pain symptoms in the leg. See Figure 5 for details.

#### Typical case 3 POD after TELD

**Figure 6:**
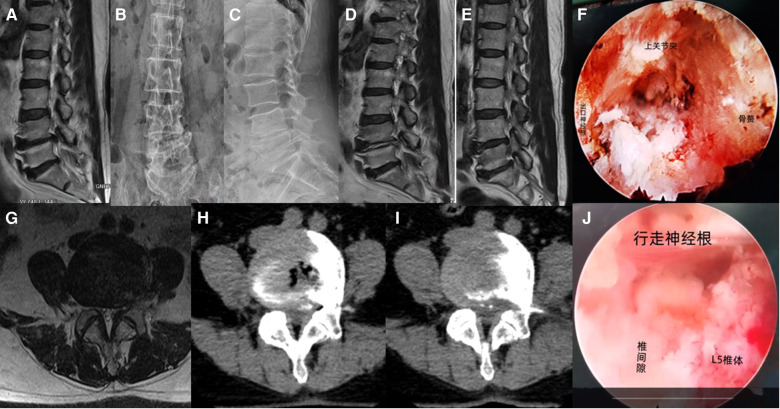
(**A**) preoperative MRI in sagittal position; (**B,C**) preoperative x-ray radiograph in lumbar dynamic position showing no lumbar instability or spondylolysis, the left L4/5 space collapsed and osteophytes formed; (**D**) sagittal MRI 2 days after surgery; (**E**) sagittal MRI 6 years after surgery; (**F**) the situation under the endoscope when inserting the working channel; (**G**) preoperative MRI in coronal position; (**H**) preoperative CT in coronal position; (**I,K**) coronal CT 2 days after surgery; (**J**) sufficient decompression of the nerve root was seen under the endoscope.

A 64-year-old female patient was admitted to the hospital with left leg pain for more than 6 months. When performing L4/5 TELD, the patient said numbness in the left anterior thigh at the time of puncture to the target, considering the export root irritation, the working channel was inserted for exploration. Under the microscope, the left L4 nerve root was significantly moved down due to the collapse of the vertebral space, and the left foraminal stenosis was caused by osteophyte hyperplasia of the left L5 vertebral body. After the successful completion of the operation, the pain symptoms of the left leg disappeared, but the left anterior leg numbness appeared. She was given acupuncture, medium-frequency pulse and neurotrophic drug for 3 months, and recovered within 3 months after the operation. See Figure 6 for details.

## Discussion

At present, there are many reports about the short-term efficacy of TELD (1, 2, 18–20), but there are few reports on the medium- and long-term clinical evaluation of TELD for more than 5 years. Sang et al. (12) followed 62 patients after TELD for 10 years and found that 9.6% of the patients underwent open revision surgery at the same segment, and 26.6% of the patients underwent other lumbar surgery. The DH was well maintained. They considered that long-term results of TELD were favorable. Multiple experts (21–23) conducted studies for at least 5 years and found that TELD could achieve satisfactory long-term clinical results. Li et al. (13) followed 42 TELD patients for at least 7 years; 6 patients (14.29%) showed POD, only 2 patients (4.76%) showed mild sensory impairment during the last follow-up, 2 patients (4.76%) underwent revision surgery during the last follow-up period, and no instability of surgical segments was found during the last follow-up. They concluded that TELD can achieve good results after long-term follow-up, that postoperative sensory impairment was a common early complication and that limited intraoperative disc removal could well protect DH and minimize the risk of residual back pain. (See Table 6 for details.)

**Table 6:** Basic situation of Mid and long-term term outcomes (>5 years) reported in domestic and foreign literature.

Author	Year	Number of patients	Years of follow-up	Method of surgery	Outcome parameter	Complications
Sang Soo Eun	2016	62	11.22 ± 0.83	transforaminal and interlaminar	VAS-B and VAS-L, ODI, radiographic findings	/
Zhiming Tu	2017	152	6.23 ± 0.35	interlaminar	VAS, ODI, modified MacNab criteria and recurrence	Dural tears, Transient POD, hematoma and wound infection
Zhen-zhou Li	2017	134	5	transforaminal	VAS, ODI, modified MacNab criteria, Percentage of pain relief of sciatica	/
Yong Ahn	2019	298	5	transforaminal	VAS, ODI, modified MacNab criteria, Perioperative data, complications and recurrence	POD, hypesthesia or transient weakness, epidural hematoma, psoas muscle hematoma, and dural tear
Xiang Li	2021	42	7.98 ± 0.47	transforaminal	VAS-B and VAS-L, ODI, JOA, modified MacNab criteria, Radiographic parameters	Residual back pain, POD

**V**AS, visual analog scale; VAS-B, VAS for back pain; VAS-L, VAS for leg pain; ODI, Oswestry Disability Index; JOA, Japanese Orthopaedic Association; POD, postoperative dysesthesia.

In our study, the VAS, JOA and ODI scores of all patients at 3 months, 6 months, and 1 year after the operation and at the last follow-up were significantly alleviated compared with those before the operation. VAS, JOA and ODI at 6 months after operation were significantly different from 3 months after operation; VAS, JOA and ODI at 6 months after operation were not significantly different from 1 year after operation and the last follow-up. We believe that postoperative recovery from TELD may have basically stabilized at 6 months after the operation.

At present, the clinical evaluation of lumbar intervertebral disc degeneration mainly uses imaging evaluation methods, and the most commonly used is Pfirrmann grade, which is divided into 5 grades according to structure, signal intensity, distinction of nucleus and anulus, and disc height (16). Sang et al. (12) reported that DH 10 years after TELD was 81.54 ± 17.40% of that before TELD, and there was no significant difference between the two. Li et al. (13) reported that DH 7 years after TELD was 84.52 ± 5.66% of that before TELD, with no significant difference between the two. Our study found no significant difference in DH between the patients at the last follow-up and before the operation, which was consistent with the results of the above scholars. Meanwhile, we found no significant difference in the Pfirrmann grade between the patients at the last follow-up and before the operation, suggesting that TELD may have no significant effect on accelerating intervertebral disc degeneration. This has not been reported in the literature thus far.

The effect of the operation on the stability of the operative segment is also an important index for the evaluation of postoperative efficacy. Sang et al. (12) reported that no obvious lumbar instability was found 10 years after TELD, and Li et al. (13) reported that no obvious lumbar instability was found 7 years after TELD. Our study found that there was no significant difference in ROM between the last follow-up patients and those before TELD, and no obvious lumbar instability was found. We though the main reason may be the minimal disturbance of the facet joint in TELD, which may also be the main reason for the low incidence rate of ASD.

Postoperative recurrence is an inevitable problem in the simple removal of the nucleus pulposus and has also become an important reference index restricting the extensive application of this operation. Sang et al. (12) reported that the 10-year postoperative recurrence rate of TELD was approximately 9.6% (6/62). Li et al. (13) reported that the postoperative recurrence rate of TELD was approximately 4.76% (2/42) 7 years after TELD. Thomas et al. (24) reported that the TELD recurrence rate was approximately 4.76% (4/84). Anthony Yeung et al. (25) conducted at least a 5-year follow-up and found that the recurrence rate of the YESS technique was 5.1% (9/176) and that of the TESSYS technique was 10% (9/90). In our 6-year follow-up, the postoperative recurrence rate was approximately 4.68% (3/62), all of which occurred within 3 months after the operation; these findings are consistent with what Li et al. (13) and Thomas et al. (24) reported.

Postoperative complications are also a common concern of clinicians and patients. TELD complications are relatively rare, and include infection, epidural hematoma, nerve root injury, dural tear, POD, exit root stimulation and other complications (1, 13, 21) The incidence of POD is relatively high, generally more than 10% (25). In our study, 13 patients (20.97%) showed various degrees of POD, which recovered spontaneously within 3 months after the operation with oral Mecobalamin tablets and Fufang Wulingzhi Tangjiang. Three patients (4.84%) showed various degrees of muscle weakness, which recovered completely within 6 months after physiotherapy, including nourishing nerves and acupuncture. The possible reasons for POD were (1) insufficient foraminoplasty, narrow operating space, and stimulation of exit root and walking root by working channel; (2) severe nerve compression before operation and reactive nerve root edema after decompression; (3) excessive use of radiofrequency around nerve root during operation; (4) blood clot stimulation in the postoperative operation area.

## Conclusion

We believe that TELD has a satisfactory medium- and long-term effect and has no significant effect on DH, ROM or intervertebral disc degeneration, but it also inevitably has some complications, such as recurrence and POD. The consequences of these complications are generally not permanent and intraoperative operations can be refined to reduce their incidence. However, due to certain limitations of this study, such as single-center retrospective studies and selection bias, lost to follow-ups, without control group, human errors of grading and measurement, the results of this study may be biased and need to be further confirmed by multicenter randomized controlled studies and longer follow-up times.

## Data Availability

The original contributions presented in the study are included in the article/Supplementary Material, further inquiries can be directed to the corresponding author/s.
